# A Systems Biology Strategy on Differential Gene Expression Data Discloses Some Biological Features of Atrial Fibrillation

**DOI:** 10.1371/journal.pone.0013668

**Published:** 2010-10-29

**Authors:** Federica Censi, Giovanni Calcagnini, Pietro Bartolini, Alessandro Giuliani

**Affiliations:** 1 Department of Technologies and Health, Istituto Superiore di Sanità, Rome, Italy; 2 Department of Environment and Health, Istituto Superiore di Sanità, Rome, Italy; Keio University, Japan

## Abstract

Atrial fibrillation (AF), the most common cardiac arrhythmia, is associated with extensive structural, contractile, and electrophysiological remodeling. In this manuscript we re-analyzed gene expression data from a microarray experiment on AF patients and control tissues, using a new paradigm based on a posteriori unsupervised strategy in which the discrimination of patients comes out from purely syntactical premises. This paradigm, more adherent to biological reality where genes work in highly connected networks, allowed us to get both a very precise patients/control discrimination and the discovery of cell adhesion/tissue modeling and inflammation processes as the main dimensions of AF.

## Introduction

Atrial fibrillation (AF) is the most common persistent cardiac arrhythmia and also the most common cause of arrhythmia-related hospitalizations [1], [2]. It has an enormous societal impact because of its very high incidence, its clinical consequences, the difficulty of its diagnosis and management. Given that its incidence increases with age and with life expectancies, increasing in both developed and developing countries, AF is projected to become an increasing burden on most health care systems [3].

The relative risk of death for people with AF is over 20% higher per year than that of age-matched controls, with stroke accounting for the majority of that greater risk [4]. AF is also associated with extensive structural, contractile, and electrophysiological remodeling, which can sustain AF itself. Current pharmacological treatments of AF present some limits because they can be ventricular proarrhythmic and not able to prevent recurrences of AF.

The understanding of the molecular events of these remodeling processes is essential for the development of new targeted therapeutic interventions so fostering a great deal of research in the elucidation of the molecular bases of the disease.

Thus far, there has been a major focus on electrical components of the remodeling process, which has been analyzed at the molecular level by candidate gene approaches that have identified expression changes in genes encoding ion channels or calcium-handling proteins [5], [6]. Some recent studies characterized the molecular basis of AF remodeling on a more global scale, using genome-wide [7]–[10] and dedicated [11] microarrays. All these studies used the classical supervised statistical technique of hypothesis testing (detecting differentially expressed genes one by one).

This approach, while surely providing an information easily understandable to biologists who are used to think on a gene-by-gene based, can be severely biased by the high dimensionality of the microarray experiments provoking a lot of chance correlations [12]. Moreover, on a physiological standpoint, the idea of genes working independently (implicit in the supervised gene-by-gene approach) is very unrealistic [13]–[15]. This is particularly cogent in the case of cardiac arrhythmias such as atrial and ventricular fibrillation that were demonstrated to be related to the feature of multistability of cardiac tissues [16], an intrinsic property emerging from the interaction of a multiplicity of different factors. New paradigms are needed if we are to succeed in unravelling multifactorial genetic causation at higher levels of physiological function [17]. Thus we shifted to an a posteriori, unsupervised approach relying on the application of principal component analysis technique in both a clustering (oblique principal components) and spectral (component extraction of the data set having as variables the different tissues and as samples the analyzed genes) mode [18].

Beside the discovery of a relevant inflammatory component in addition to the already known cell adhesion/tissue remodeling one, our approach allowed us to confirm the ‘attractor-like’ hypothesis of gene expression regulation and demonstrated a very deterministic structure down to very minor regulation modules.

In a clinical perspective the very efficient patient/control discrimination obtained opens the way for both a quantitative estimation of disease gravity and efficacy of therapeutic interventions.

## Materials and Methods

### A. Expression data

The data were obtained from the public functional genomics data repository of the National Institute of Health (called Gene Expression Omnibus, GEO). Data from record #GSE2240 have been analyzed, consisting of samples of right atrial myocardium (appendage). Data were related to two Affymetrix platforms U133A and U133B. Right atrial appendages were obtained from 30 patients undergoing open heart surgery for valve repair or coronary artery bypass grafting. Of these, 10 patients had permanent AF defined as duration of AF longer than 3 months as documented by ECG, whereas 20 patients had no history of AF and were in SR when open heart surgery was performed. Details on clinical protocol and hybridization procedures can be found in [10], [13]. According to these previous publication, all patients gave written informed consent.

### B. Data analysis strategy

The data are organized in such a way to have the genes as statistical units and the patients as variables. The genome-wide expression profiles of each individual (variables), both AF and control subjects, were clustered by a divisive clustering algorithm based on Oblique Principal Component Analysis (OPCA, [19]). The original data set is progressively subdivided in clusters with the goal to cluster together maximally correlated variables. The progressive division of the data set corresponds to the generation of clusters of variables and to generate clusters the more independent of each other: this is a maximum intra cluster correlation/minimal inter clusters correlation criterion analogous to the k-means procedure [19]. In this case, given the huge between variables (genome wide profiles) correlation, the system gives by default a single cluster solution (all the profiles pertaining to the same cluster) explaining the 98.7% of total variance. This result is consistent with the well known fact that any sample of a particular tissue has a strongly invariant gene expression profile. To check if an unsupervised clustering strategy was consistent with normal vs pathology classification, we set a priori the number of clusters: we forced the software to generate a two cluster solution whose relative asymmetry in control/patients composition is thus a completely unbiased (no choice of genes, no a priori driving of solution) measure of discrimination. This analysis was followed by a principal component analysis (PCA) of the data set.

The principal components were extracted from the same matrix. Patients and controls are then defined in the space of component loadings which represent different individuals in terms of similarities in the gene expression space. The loading space was then analysed by a linear discriminant analysis (a supervised procedure) based on three components (two from U133A and one from U133B space) allowing for an almost perfect (only one misclassified unit) separation of the data set into patients and controls. PCA defines single genes in the space of component scores, allowing for a biological association of components to groups of genes having the highest absolute scores and thus permitting a biological interpretation of the obtained discrimination.

## Results

OPC analysis on the U133A data set generated an optimal two cluster solutions of the data set exhibiting the composition in terms of control and disease samples reported in Table 1.

**Table 1:** Optimal cluster solution generated by OPC analysis on the U133A data set.

	Control	AF patients
Cluster1a	15	0
Cluster2a	5	10

The same procedure as applied to U133B set generated the contingency table reported in Table 2.

**Table 2:** Optimal cluster solution generated by OPC analysis on the U133B data set.

	Control	AF patients
Cluster1b	3	16
Cluster2b	7	4

Both the classifications are significantly related to the patient/control discrimination scoring a Fisher's exact test significance equal to p<0.0001 and p<0.015 respectively.

This points to a global, genome-wide, significant discrimination of the two groups. In order to go in depth and refining this preliminary ‘raw’ result, we separately applied PCA to the two U133A and U133B sets.

Tables 3 and 4 show proportional and cumulative variance expressed by the first 10 PCs, for microarray data extracted from chip U133A and U133B, respectively.

**Table 3:** Proportional and cumulative variance expressed by the first 10 PCs, for microarray data extracted from chip U133A.

Eigenvalue	Proportionn	Cumulative
1	0.9842	0.9842
2	0.0032	0.9874
3	0.0021	0.9895
4	0.0014	0.9909
5	0.001	0.9919
6	0.0009	0.9928
7	0.0007	0.9935
8	0.0007	0.9941
9	0.0006	0.9947
10	0.0005	0.9952

**Table 4:** Proportional and cumulative variance expressed by the first 10 PCs, for microarray data extracted from chip U133B.

Eigenvaluee	Proportionn	Cumulative
1	0.9835	0.9835
2	0.0035	0.9870
3	0.0028	0.9898
4	0.0010	0.9908
5	0.0008	0.9917
6	0.0008	0.9924
7	0.0006	0.9930
8	0.0006	0.9936
9	0.0005	0.9942
10	0.0005	0.9946

**Figure 1:**
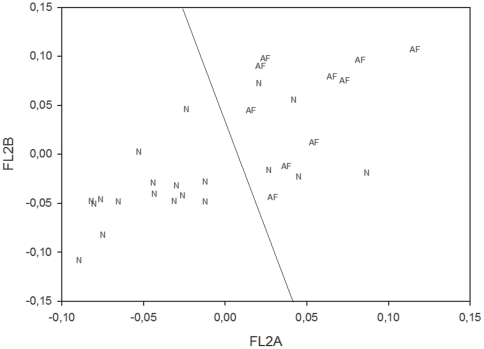
The graph axes are the genome-wide profiles of two samples (an AF and a control one), the vector points are the single genes. The overwhelming order parameter correlating around 20000 genes expression values is evident. The line roughly corresponds to PC1.

The first PC, for each chip, accounts for more than 98% of the total variability so pointing to a remarkable general similarity between samples' profiles as evident in Fig. 1.

A lot of experimental evidences [20]–[21] point to the genome regulation as the dynamics of an highly connected system that cannot be profitably *a priori* factorized into single genes independent dynamics. This connectivity is at the basis of the consideration of cell kinds as ‘attractors’ in multidimensional spaces constituted by the characteristic expression values of the different genes [21]. This attractor-like (and very deterministic) properties of gene expression hold at the cell population level, while, at the single cell level, stochasticity seems to prevail [21]. Since the population level is the one important for our analysis that deals with tissue properties, the analysis of the genome profiles as a whole is of utmost importance for the description of between-lines differences. The presence of a very strong common attractor correspondent to the specific tissue and cell kind leads to between-samples correlations close to one, as for the genome-wide expression profile. Globally the commonality between gene expression profiles on the genome-wide scale accounts for 98% of total variability in both sets. This overwhelming commonality confines the between-samples differences into minor components. The principal component analysis (PCA) projects by construction the initial space spanned by the different samples into a new derived space whose axes (principal components) are each other orthogonal. It allows for a direct, unbiased normalization of the data field, where the ‘shared variance’ is accounted for by the first principal component (attractor) and the minor components (from second component onward) keep trace of the relevant among samples differences.

The analysis of the factor loadings (FL, correlation coefficients between original variables and components) of the first 6 PCs revealed that the FLs which better discriminate between permanent AF patients and controls were: FL of the 2^nd^ PC for both chips (namely, FL2A and FL2B); FL of the 3^rd^ component of chip A (FL3A); and FL of the 5^th^ component of chip B (FL5B). A linear discriminant analysis (LDA) based on the combination of these 4 FLs leads to the classification reported in table 5.

**Table 5:** Classification of patients combining FL2A, FL2B, FL3A and FL5B by LDA.

	AF	Control	Total
AF	10	0	10
Control	1	19	20
Total	11	19	

The classification obtained by LDA on the four selected components space is extremely accurate with only one missed sample.

**Figure 2:**
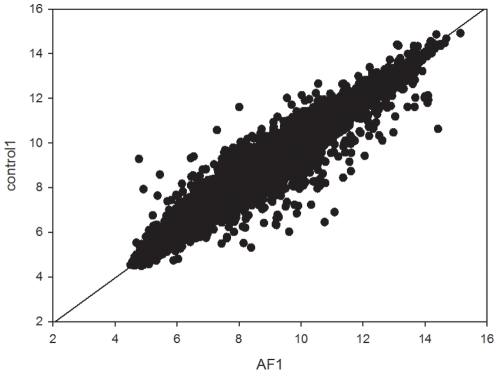
Discrimination plane obtained combining FL2A and FL2B. The points in the plot correspond to individual patients (N = normal patients; AF =  atrial fibrillation patients). The plot is spanned by the two most relevant discriminating factors obtained from the two chips. The discriminating line is the result of the application of linear discriminant analysis on the FL2A and FL2B space.

Figure 2 shows the discrimination plane obtained combining FL2A and FL2B. This reduced bidimensional plane does not allow to get the same accuracy as the four dimensional one used for LDA, but it explains the general logic of the method.

The correlation of each FL of one chip with the corresponding FL of the other chip leads to the results reported in table 6. Surprisingly, the FL of one chip turned out to be highly correlated with the corresponding one of the other chip up to the 6^th^ PC. The surprise comes from the fact that, even if the minor components are more and more affected by noise [19], we found relevant correlations for components like the sixth, which explains as few as 8 parts out of ten thousands of the global variability. It suggests an extremely deterministic type of control. Clearly this determinism, analogously to the strict determinism of thermodynamic laws, arises as an average over millions of single stochastic elements (cells). This tissue level control is probably at the basis of the organ reliability [14], [15], [21]. This finding is extremely relevant considering U133A and U133B share only a minimal portion of common genes. The fact that U133B has a larger portion of not annotated genes than U133A is a further proof of the fact that genomes work as a network rather than as a summation of the activities of independent units. This implies that any sufficiently wide sampling of the genome is an almost equivalent system.

**Table 6:** Correlation among the FLs of the two chips (U133A and U133B).

	FL1A	FL2A	FL3A	FL4A	FL5A	FL6A
FL1B	**0.70**	0.01	*0.38*	−0.39	−0.21	−0.12
FL2B	0.01	**0.72**	−*0.59*	−0.10	−0.12	0.13
FL3B	0.15	−0.38	**−0.64**	−0.21	−0.02	0.14
FL4B	−0.44	−0.28	−0.32	**0.80**	−0.10	0.15
FL5B	−0.26	0.18	−0.14	0.31	**0.75**	−0.23
FL6B	−0.13	−0.11	0.11	−0.04	0.11	**0.70**

The genes having the highest 50 scores (in module) of the discriminant components were extracted to give a biological meaning of the observed patient/control discrimination. Table 7 shows the genes with the highest scores (in absolute value) for the factor loadings of the 2^nd^ PCs for chip U133A (FL2A).

**Table 7:** Genes with the highest scores (in absolute value) for the factor loadings of the 2^nd^ PCs for chip U133A (FL2A).

Gene	Gene Ontology
**NPPB**	**natriuretic peptide precursor B**
**CHGB**	**chromogranin B (secretogranin 1)**
DHRS9	dehydrogenase/reductase (SDR family) member 9
**IGFBP2**	**insulin-like growth factor binding protein 2**
**COLQ**	**collagen-like tail subunit (single strand of homotrimer) of asymmetric acetylcholinesterase**
**LTBP2**	**latent transforming growth factor beta binding protein 2**
**LRRC2**	**leucine rich repeat containing 2**
**HSPA2**	**Heat shock 70kDa protein 2**
EIF1AY	eukaryotic translation initiation factor 1A
**PHLDA1**	**pleckstrin homology-like domain, family A, member 1**
IGF2	insulin-like growth factor 2 (somatomedin A)
RPS4Y1	ribosomal protein S4, Y-linked 1
**EIF5A**	**eukaryotic translation initiation factor 5A**
**TNC**	**tenascin C**
**ASPN**	**asporin**
**FRZB**	**frizzled-related protein**
LOXL2	lysyl oxidase-like 2
MUC5AC	mucin 5AC, oligomeric mucus/gel-forming
**FRZB**	**frizzled-related protein**
**ETV5**	**ets variant 5**
**PHLDA1**	**pleckstrin homology-like domain, family A, member 1**
THBS4	thrombospondin 4
EIF1AY	eukaryotic translation initiation factor 1A
NCRNA00185	non-protein coding RNA 185
PFKFB2	6-phosphofructo-2-kinase/fructose-2,6-biphosphatase 2
**TNNI1**	**troponin I type 1 (skeletal, slow)**
**PIK3R1**	**phosphoinositide-3-kinase, regulatory subunit 1 (alpha)**
TTC3	tetratricopeptide repeat domain 3
TTC3	tetratricopeptide repeat domain 3
MARCH6	membrane-associated ring finger (C3HC4) 6
LYVE1	lymphatic vessel endothelial hyaluronan receptor 1
**TPR**	**translocated promoter region (to activated MET oncogene)**
**NTRK2**	**neurotrophic tyrosine kinase, receptor, type 2**
**NRIP1**	**nuclear receptor interacting protein 1**
SFRP1	secreted frizzled-related protein 1
**STAG2**	**stromal antigen 2**
**GPM6B**	**glycoprotein M6B**
PHACTR2	phosphatase and actin regulator 2
**ALDH1A1**	**aldehyde dehydrogenase 1 family, member A1**
RBP4	retinol binding protein 4, plasma
**INHBA**	**inhibin, beta A**
CTSZ	cathepsin Z
IGH	immunoglobulin heavy locus
SFRP1	secreted frizzled-related protein 1
**SLIT2**	**slit homolog 2 (Drosophila)**
JUN	jun oncogene
CEP350	centrosomal protein 350kDa
VEZF1	vascular endothelial zinc finger 1
XIST	X (inactive)-specific transcript (non-protein coding)
TNPO1	transportin 1

The genes reported bold are those previously found on the same data by the group who perform the atrial biopsy in 2005.

The genes reported in gray rows are those previously found on the same data by the group who perform the atrial biopsy in 2005, using the classical methods of gene up- and down-expression of patients respect to controls [10], [12]. The large number of genes in common extracted with two completely different methodologies is a further proof of the robustness of the adopted strategy.

Looking at the biological functions of the genes most influenced by the discriminant components we can try and give a functional characterization to the obtained discrimination. With some exceptions of genes specifically linked to heart functioning (natriuretic factor), the great majority of the extracted genes pertains to two main ‘biological classes’: genes linked to tissue organization and heart structure and genes involved in inflammatory processes.

## Discussion

The mechanism of AF in human tissues is extremely complex, because atrial remodeling consists of electrical, contractile, and structural remodeling. In addition, structural remodeling may occur from chronic hemodynamic, metabolic, or inflammatory stressors. The cellular and molecular basis of AF is a field of enormous interest. Many factors such as ion channels, proteins influencing calcium homeostasis, connexins, autonomic innervation, fibrosis, and cytokines may be involved in the molecular mechanism of AF.

Some aspects of the molecular mechanisms underlying the genetic variability of AF and the perioperative cardiovascular risk have been investigated, indicating the alteration of genes involved in oxidative stress, inflammation and coagulation [10], [11], [22]–[24].

In this paper we applied the PCA to microarray data obtained from permanent AF patients and no-AF control group. The underlying hypothesis of the strategy is that the AF signature in terms of differential gene expression cannot be traced back to the independent activation of single players (genes) but on a general modulation of the entire genome.

Comparing our methodology with the commonly used general supervised inferential approach (with statistical test such as SAM) we were able to better characterized different physiopathological aspects of AF, aspects impossible to be separately identified by a classical supervised approach [12].

Unlike the classical meta-analysis approach [25] which tries to identify sets of relevant genes shared by independent studies, we abandoned the concept of the selection of important genes as main goal of the procedure, to shift toward an unsupervised approach centered on the elucidation of major fluxes of gene expression correlations as defined by principal component analysis.

As expected, the first PC accounts for more than 98% of the total population variability; the first PC can be considered as the common substrate of each individual myocardium. The differences in gene expression profiles between permanent AF patients and controls are related to a very small part of the data variability. However, the analysis of such a small difference in terms of factor loadings and scores, succeed in discriminating patients from controls and extracting further genes involved in the pathology, respect to those already detected. The strict deterministic character (at the population scale) of the fine modulation correspondent to the minor components is proven by the strong correlations existing between partially independent gene expression panels.

A careful investigation of the genes endowed with highest scores relative to the second component of gene expression (the only component common to the two U133A and U133B endowed with an elevated discriminant power) reveals groups of genes that are involved in cardiac muscle structure and organization and in inflammatory processes. Most of these genes are known to be markers of the pathology, validating our approach; however, we detect further genes involved in the pathology that allows completing the transcriptomic deregulation picture of the pathology studied.

It is worth noting the virtual absence of genes directly involved in the generation of the electrical stimulus at the single ion channel microscopic level. This is a very important point that in some way alters the classical picture of the disease: the most relevant information for the AF disease are located at the level of tissue organization that in turn is linked to the stimulus conduction and generalization on the tissue scale and not at the level of single cell stimulus onset.

Both tissue organization [26] and inflammation [27] are well known players in atrial fibrillation, thus our analysis gave results consistent with the clinical evidences. It is worth noting that both tissue organization and inflammation are systemic features hardly decomposable into single genes contributions [17].

At this level of analysis is practically impossible to separate ‘causes’ from ‘consequences’, i.e. modifications in gene expression that can play a role in the onset of the pathology and modifications that are induced by the fibrillation event.

We hope further experimentation along this way could shed light into this very important point.

### Conclusion

This manuscript applies a novel approach for the processing of microarray data of atrial tissue in persistent AF patients. This approach allows a clear discrimination between microarray expression profiles of persistent AF patients respect to a control population. The analysis of genes involved in this clustering reveals modification of microarray expression in genes involved in cardiac muscle structure and organization and in inflammatory processes.
