# Correction: A Systems Biology Strategy on Differential Gene Expression Data Discloses Some Biological Features of Atrial Fibrillation

**DOI:** 10.1371/annotation/49c9c072-6584-4b6d-a636-1b15f6a76f4d

**Published:** 2010-11-17

**Authors:** Federica Censi, Giovanni Calcagnini, Pietro Bartolini, Alessandro Giuliani

The images for Figures 1 and 2 were incorrectly switched. The image that appears as Figure 1 should be Figure 2, and the image that appears as Figure 2 should be Figure 1. The figure legends appear in the correct order.

